# Adiponectin is not required for exercise training‐induced improvements in glucose and insulin tolerance in mice

**DOI:** 10.14814/phy2.12146

**Published:** 2014-09-11

**Authors:** Ian R. W. Ritchie, David C. Wright, David J. Dyck

**Affiliations:** 1 Department of Human Health and Nutritional Sciences, University of Guelph, Guelph, Ontario, Canada

**Keywords:** Adiponectin, adiponectin knockout mice, exercise training, glucose and insulin tolerance, insulin response, skeletal muscle

## Abstract

Adiponectin (Ad) is a potent insulin‐sensitizing adipokine that has been found to activate pathways involved in the adaptation to exercise. Therefore, we examined whether Ad is required for the increased insulin response observed following exercise training in Ad knockout mice (AdKO). Eight weeks of exercise training significantly increased glucose and insulin tolerance in both wild type (WT) and AdKO mice. There were no differences in glucose tolerance between genotypes but insulin tolerance was improved to a greater extent in AdKO compared to WT mice following exercise training (+26%, *P* < 0.05). There were no genotype differences in the insulin‐stimulated phosphorylation of AKT or AS160 in red or white gastrocnemius muscle (RG, WG). Exercise training increased total AKT and AS160 protein content in RG and total AS160 protein content in WG. There were no genotype differences in total AKT or AS160. However, exercise training induced a more robust increase in total AS160 in RG from AdKO (+44 ± 8%, *P* < 0.05) compared to WT mice (+28 ± 7%, *P* = 0.06). There were no differences in total GLUT4 or FAT/CD36 in RG or WG in WT or AdKO, with or without exercise training. Similarly, there were no differences in RER, VO_2_, or activity between any groups. Our results indicate the presence of Ad is not required for exercise‐induced increases in insulin response. Furthermore, it appears that exercise may improve insulin sensitivity to a greater extent in the absence of Ad, suggesting the presence of an unknown compensatory mechanism.

## Abstract (precis)

Collectively, our results demonstrate that the absence of Ad does not impair the capacity of endurance exercise training to increase glucose and insulin tolerance in AdKO mice. In addition, there were no impairments in insulin signaling or in the protein content of AKT or AS160. Taken together with previous findings, our data indicate that AdKO mice may have sufficient compensations to override the absence of Ad.

## Introduction

Since the discovery of adiponectin (Ad), a large body of evidence has established its role as a potent regulator of hepatic and skeletal muscle insulin sensitivity (Berg et al. 2001; Combs et al. 2001; Yamauchi et al. 2001, 2002). Circulating Ad is strongly correlated with insulin sensitivity (Hotta et al. 2001) and is reduced in obese and diabetic animal models and humans (Arita et al. 1999; Hotta et al. 2001; Yamauchi et al. 2001). Regular treatment with Ad increases insulin sensitivity in lean mice and ameliorates insulin resistance in diet‐induced and genetically obese mice (Yamauchi et al. 2001). This response has been attributed in part to the activation of AMP‐activated protein kinase (AMPK) and the subsequent increase in fatty acid (FA) oxidation and a reduction in intramuscular lipids (Yamauchi et al. 2002). Adiponectin treatment has also been shown to directly increase insulin signaling in C2C12 myotubes by reducing the activity of p70 S6 kinase, thereby reducing the inhibition of IRS‐1 in response to insulin (Wang et al. 2007). Finally, the ability of the thiazolidinedione (TZD), pioglitazone, to improve whole‐body insulin sensitivity is markedly compromised in mice deficient in Ad (Kubota et al. 2006).

An increase in insulin sensitivity is a hallmark response to exercise training (Wallberg‐Henriksson et al. 1988; Cartee et al. 1989; Host et al. 1998; Wojtaszewski et al. 2000; Frosig et al. 2007). This appears to be mediated by a combination of factors including an increase in the expression or activity of proteins involved in insulin signaling and glucose transport (Chibalin et al. 2000; Frosig et al. 2007), an increase in oxidative capacity (Bruce et al. 2003), improvement in lipid handling/storage (Liu et al. 2007; Schenk and Horowitz 2007), and weight loss (Fruebis et al. 2001; Yamauchi et al. 2001). Skeletal muscle response to exercise is largely attributed to local signaling that accompanies muscle contraction. This includes perturbations to the ratio of AMP to ATP and intracellular Ca^2+^ which regulate insulin sensitivity and mitochondrial biogenesis through the activation of CAMK (Rose and Hargreaves 2003; Smith et al. 2007; Wright et al. 2007) and AMPK (Winder et al. 2000; Ojuka et al. 2002; Sriwijitkamol et al. 2007). Some, however, have questioned the notion that skeletal muscle adaptations to exercise are strictly due to autonomous signaling. Skeletal muscle insulin sensitivity is increased immediately following acute exercise or in situ contractions (Cartee and Holloszy 1990; Gao et al. 1994). However, if a muscle is contracted in isolation ex vivo, an acute increase in insulin sensitivity occurs only in the presence of serum, suggesting the need for an unknown circulating factor (Cartee and Holloszy 1990; Gao et al. 1994). Little progress has been made in identifying said factor.

Recently, it has been demonstrated that exercise‐induced mitochondrial biogenesis does not occur in ob/ob mice (Li et al. 2011), prompting the authors to suggest that intact leptin and adiponectin signaling is required for this adaptation. This is supported by the findings that obese and diabetic humans, who have reduced circulating Ad and are generally leptin and Ad resistant, have diminished phosphorylation of AMPK in response to exercise (Sriwijitkamol et al. 2007). There is considerable overlap between the response of skeletal muscle to exercise and Ad. Like exercise, Ad activates Ca^2+^ and AMPK signaling pathways (Iwabu et al. 2010) and increases mitochondrial content (Iwabu et al. 2010) and insulin sensitivity (Yamauchi et al. 2001). Adiponectin had been found to increase with endurance exercise (Kriketos et al. 2004). Interestingly, skeletal muscle AMPK activity from obese humans is resistant to activation by Ad (Bruce et al. 2005). It is conceivable then, that Ad could mediate some of the effects of exercise training. However, we have recently shown that exercise‐induced increases in mitochondrial protein expression are indistinguishable in skeletal muscle from WT and AdKO mice (Ritchie et al. 2014). Although this strongly suggests that Ad is not necessary for mitochondrial adaptations to exercise training, it is possible that Ad could be required for exercise training‐ induced improvements in insulin sensitivity. Therefore, the purpose of this study was to determine if improvements in whole body glucose and insulin tolerance induced by exercise training, are dependent on the presence of Ad.

## Methods

### Housing and diets

Male wild‐type (WT, C57BL/6J) and adiponectin knockout (AdKO, B6.129‐Adipoqtm1Chan/J) mice from The Jackson Laboratory (Bar Harbor, ME) were housed in pairs, were given ad libitum access to standard rodent chow (Harlan Teklad, Madison, WI) and were maintained on a 12‐h light‐dark cycle. At approximately 12 weeks of age, WT and AdKO animals were randomly assigned to exercise‐trained and untrained (sedentary) groups. All procedures were approved and ethical consent was provided by the Animal Care Committee at the University of Guelph.

### Chronic exercise protocol

Trained animals were exercised on a treadmill 5 days per week for 8 weeks. Mice ran at 20 m/min for the first 3 weeks for 45 min, with the incline increasing from 5% (week 1) to 15% (week 3). The incline was held constant at 15% for the remaining 5 weeks, and the speed increased to 25 m/min by week 5. Exercise duration was increased to 60 min for the last 3 weeks. During the last 2 weeks of training, 30‐sec sprints (32 m/min) were performed at 10 min intervals. Exercise‐trained animals were given a 48 h rest period after the last training bout before performing experimental procedures in order eliminate the effects of the last exercise bout (acute effects).

### Glucose and insulin tolerance tests

Following 8 weeks of experimental treatment, exercise‐trained mice, and their respective controls were subjected to intraperitoneal glucose (6 h fast; 2 g/kg) and insulin (fed; 0.75 U/kg body weight) tolerance tests. Tail vein measurements of blood glucose were determined with a handheld glucometer (Freestyle Lite, Abbott Diabetes Care Inc., Alameda, CA). Tolerance testing was separated by a minimum of 3 days.

### Clams

Metabolic monitoring (nonexercise) was performed using a Comprehensive Lab Animal Monitoring System (CLAMS, Columbus Instruments, Columbus, OH). A challenge with this system is that in isolation and in an unfamiliar environment, animals occasionally fast voluntarily with obvious implications for metabolism. To help the animals acclimate, they were placed in the metabolic caging for 18–24 h 1 week before data collection. Additionally, animals were acclimatized for 4–6 h in the metabolic caging unit immediately prior to data collection. Data (RER, VO_2_, activity) were collected over a 24 h period and averaged over the light and dark periods separately.

### Surgical procedures and insulin signaling

48 h after the last exercise bout, sedentary and exercise‐trained mice were anaesthetized with an intraperitoneal injection of sodium pentobarbital (6 mg/100 g body wt). Red and white gastrocnemius were sampled from one hind limb and immediately frozen in liquid nitrogen, at which time animals were given an intraperitoneal injection of insulin (10 U/kg). Ten min post injection, red and white gastrocnemius were sampled and immediately frozen in liquid nitrogen. Tissues were stored at −80°C for subsequent use in western blotting.

### Western blot analyses

Muscle samples were homogenized in an ice‐cold buffer for the extraction of proteins and preservation of protein phosphorylation states. The buffer contained 50 mmol/L Tris (pH = 7.5), 1 mmol/L EDTA, 1 mmol/L EGTA, 50 mmol/L NaF, 5 mmol/L sodium pyrophosphate, 10% (vol/vol) glycerol, 1%(vol/vol) Triton X‐100, 2 mg/mL leupeptin, 2 mg/mL aprotinin, 2 mg/mL pepstatin, 1 mmol/L dithiothreitol, and 1 mmol/L phenylmethylsulfonyl fluoride. Muscle homogenates were sonicated and centrifuged at 1500 ×* g* for 20 min at 4°C and the supernatant removed and protein content determined via BCA assay. Fifteen micrograms of whole muscle tissue lysate protein was solubilized in 4 × Laemmeli's buffer and boiled at 95°C for 10 min, resolved by SDS‐PAGE, and wet transferred to PVDF membranes for 1 h at 100 V. The membranes were blocked with 5% BSA for 2 h and then incubated with the specific primary antibodies for adiponectin, GLUT4 (#'s ab22554, ab654, respectively; Abcam Inc., Cambridge, MA), FAT/CD36 (#sc‐13572; Santa Cruz Biotechnology Inc., Dallas, Tx)**,** total AKT, pAKT serine 473, pAKT threonine 308, total AS160, pAS160 serine 318, pAS160 serine 588 (#'s4685, 4060, 4056, 2670, 8619, 8730 respectively; Cell Signaling, Danvers, MA) overnight. After incubation with the appropriate secondary antibody, the immune complexes were detected by enhanced chemiluminescence and were quantified by densitometry (Fluorochem HD2, Protein Simple, Toronto, ON, Canada). Alpha tubulin (ab7291, Abcam) was used to ensure consistent protein loading and transferring.

### Statistical analysis

All data are reported as mean ± the standard error (SE). Data were analyzed using a combination of two and three‐way analysis of variance (ANOVA). A two‐way ANOVA was used to determine if there were significant differences in glucose and insulin tolerance, GLUT4, FAT/CD36, AKT, and AS160 protein content that could be attributed to genotype or exercise training. A three‐way ANOVA was used to determine if there were significant differences in the phosphorylation of AKT and AS160 that could be attributed to insulin treatment, genotype, or exercise training. Results from the ANOVAs were assessed by Student‐Newman‐Keul's post hoc test. Significance was accepted with a *P* value ≤ 0.05.

## Results

### Serum and tissue adiponectin content

The absence of adiponectin in muscle (red gastrocnemius), adipose tissue (eWAT), and serum was confirmed and reported previously (Ritchie et al. 2014).

### Body weights

There were no differences in body weight between WT and AdKO mice with or without exercise training (Table 1).

**Table 1.** Comprehensive lab animal monitoring system (CLAMS) data, terminal body weights and fasting blood glucose from sedentary and exercise‐trained wild‐type and AdKO mice.

	WT‐Sed		WT‐Ex		AdKO‐Sed		AdKO‐Ex	
	Light	Dark	Light	Dark	Light	Dark	Light	Dark
RER	0.84 ± 0.01	0.92 ± 0.01	0.84 ± 0.01	0.93 ± 0.01	0.84 ± 0.01	0.92 ± 0.01	0.86 ± 0.01	0.93 ± 0.01
VO_2_ (mL/min/kg)	48 ± 4	56 ± 5	49 ± 3	55 ± 4	47 ± 4	52 ± 4	51 ± 2	58 ± 2
Activity (counts)	56 ± 8	197 ± 33	80 ± 12	258 ± 31	60 ± 17	212 ± 48	96 ± 13	250 ± 28
Body weight (g)	28.7 ± 0.6		28.3 ± 0.5		28.7 ± 0.6		28.5 ± 0.4	
Fasting glucose (mmol/L)	7.7 ± 0.2		7.8 ± 0.3		7.5 ± 0.4		8.1 ± 0.3	

Data are expressed as the mean ± SE, *n* = 8–10.

### RER, VO_2_, and activity

There were no differences in RER, VO_2_, or activity values between WT and AdKO mice or between sedentary and exercise‐trained mice. This was true for both light and dark cycles (Table 1).

### Intraperitoneal glucose and insulin tolerance tests

**Figure 1.**
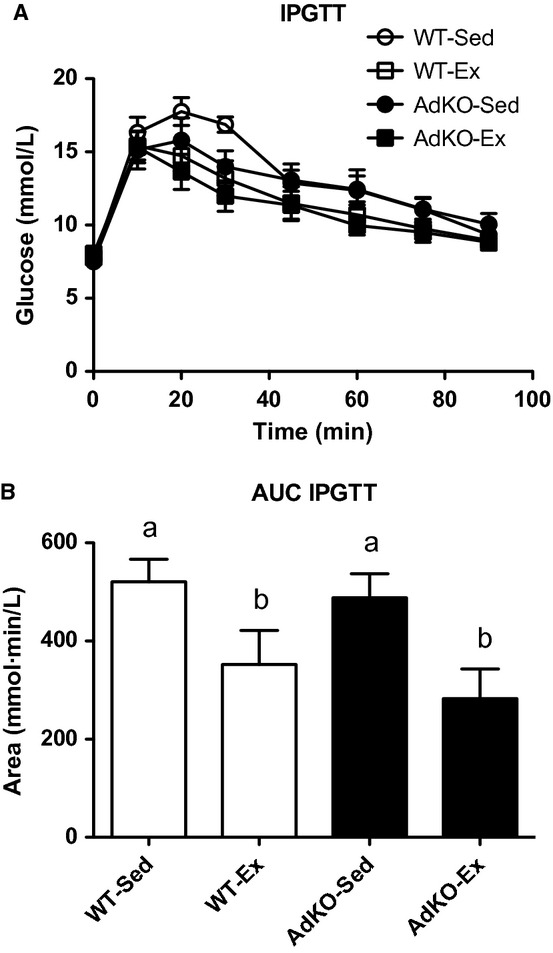
Blood glucose (A) and the area under the blood glucose curve (B) measured during an intraperitoneal glucose tolerance test. Data are expressed as mean ± SE,* n* = 10. Experimental groups not sharing a letter are significantly different, *P *< 0.05. WT‐Sed, wild‐type+no exercise; WT‐Ex, wild‐type+exercise training; AdKO‐Sed, Ad knockout+no exercise; Ad‐Ex, Ad knockout+exercise training.

There were no differences in fasting blood glucose between experimental groups (Table 1). There were no differences in response to glucose injection amongst groups except within the sedentary animals at 30 min (WT‐Sed, 16.8 ± 0.5; AdKO‐Sed, 14.0 ± 0.9 mmol/L; *P* < 0.05). The calculated incremental area under the curve (AUC) for glucose tolerance was significantly reduced, that is, improved with exercise training in both WT and AdKO mice, with no differences between genotypes (Fig. 1).

**Figure 2.**
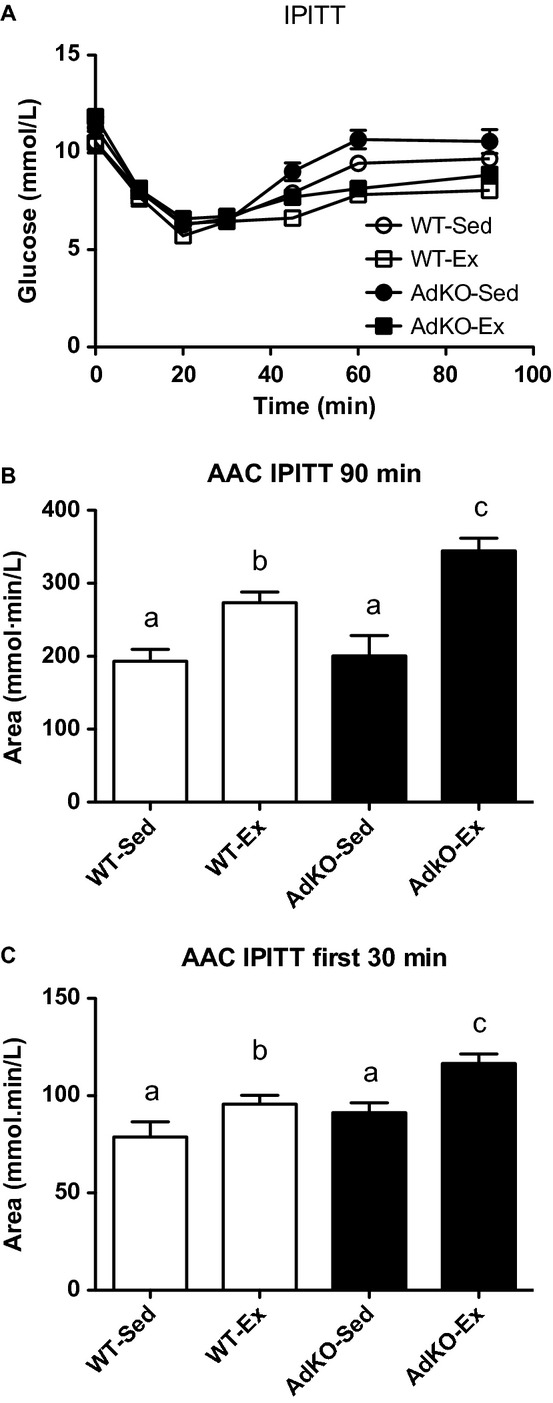
Blood glucose (A), the area over the entire blood glucose curve (B) and the area over the blood glucose curve strictly during the first 30 min measured during an intraperitoneal insulin tolerance test. Data are expressed as mean ± SE,* n* = 6. Experimental groups not sharing a letter are significantly different, *P *< 0.05. WT‐Sed, wild‐type+no exercise; WT‐Ex, wild‐type+exercise training; AdKO‐Sed, Ad knockout+no exercise; Ad‐Ex, Ad knockout+exercise training.

There were no significant differences in blood glucose in response to insulin between the groups within the first 30 min. There was an overall exercise training and genotype effect at all subsequent time points. At 45 and 60 min blood glucose was significantly reduced with training in AdKO mice but not WT mice (45 min: AdKO‐Sed, 9.0 ± 0.5; AdKO‐Ex, 7.7 ± 0.3 mmol/L; 60 min: AdKO‐Sed 10.6 ± 0.5; AdKO‐Ex .2 ± 0.4 mmol/L; *P* < 0.05). At 75 min, blood glucose was significantly reduced with exercise training in both WT and AdKO mice (WT‐Sed, 9.7 ± 0.2; WT‐Ex, 8.1 ± 0.2; AdKO‐Sed, 10.6 ± 0.6; AdKO‐Ex, 8.9 ± 0.4 mmol/L; *P* < 0.05). The area above the curve was increased with exercise training in both WT and AdKO mice (i.e., greater glucose clearance in response to insulin). There were no differences in area between sedentary groups; however, the area for exercise‐trained AdKO mice was significantly greater than that of exercise‐trained WT mice. Area above the curve was also calculated over the first 30 min to better discern the effects on insulin on glucose disposal independently from the mobilization of endogenous glucose apparent in the latter portion of the insulin tolerance test. This yielded similar, although less pronounced effects with respect to exercise training (Fig. 2).

### Total and phosphorylated AKT and AS160

**Figure 3.**
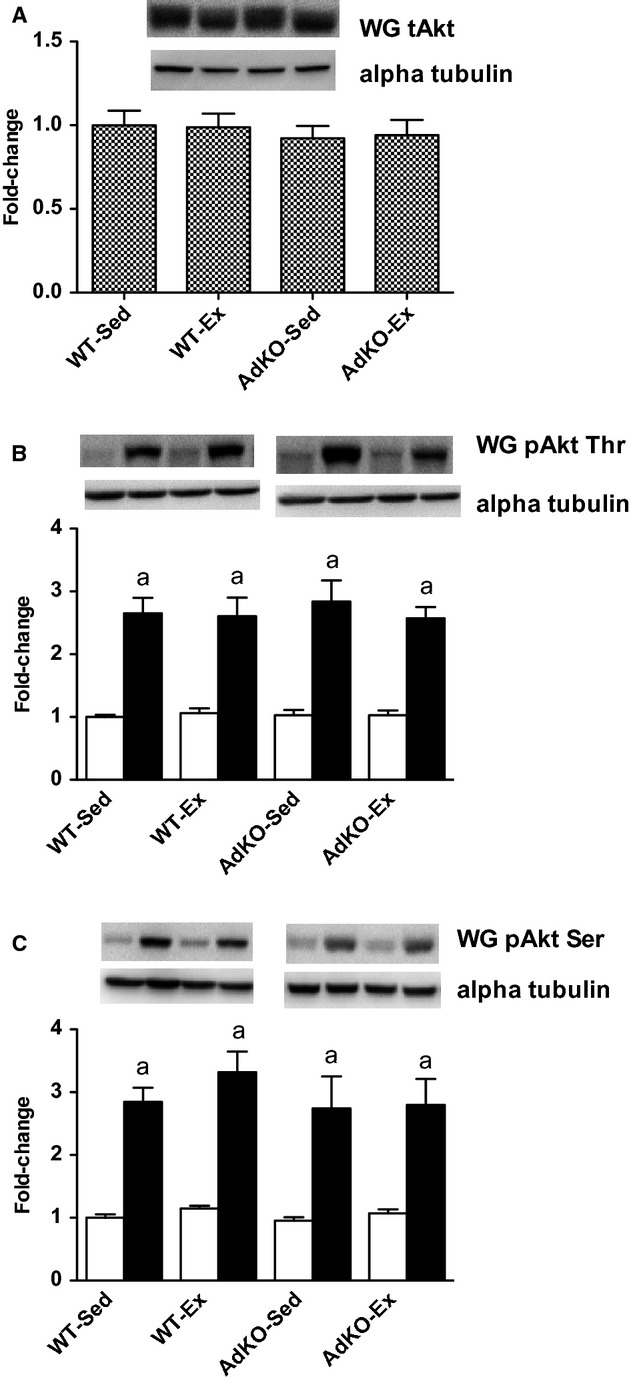
Western blots of total AKT protein content (A) and basal (open bars) and insulin‐stimulated (filled bars) phosphorylation of AKT at threonine 308 (B) and serine 473 (C) from white gastrocnemius muscle. Data are expressed relative to basal WT‐Sed group, mean ± SE,* n* = 8–10. Experimental groups not sharing a letter are significantly different, *P *< 0.05. WT‐Sed, wild‐type+no exercise; WT‐Ex, wild‐type+exercise training; AdKO‐Sed, Ad knockout+no exercise; Ad‐Ex, Ad knockout+exercise training.

**Figure 4.**
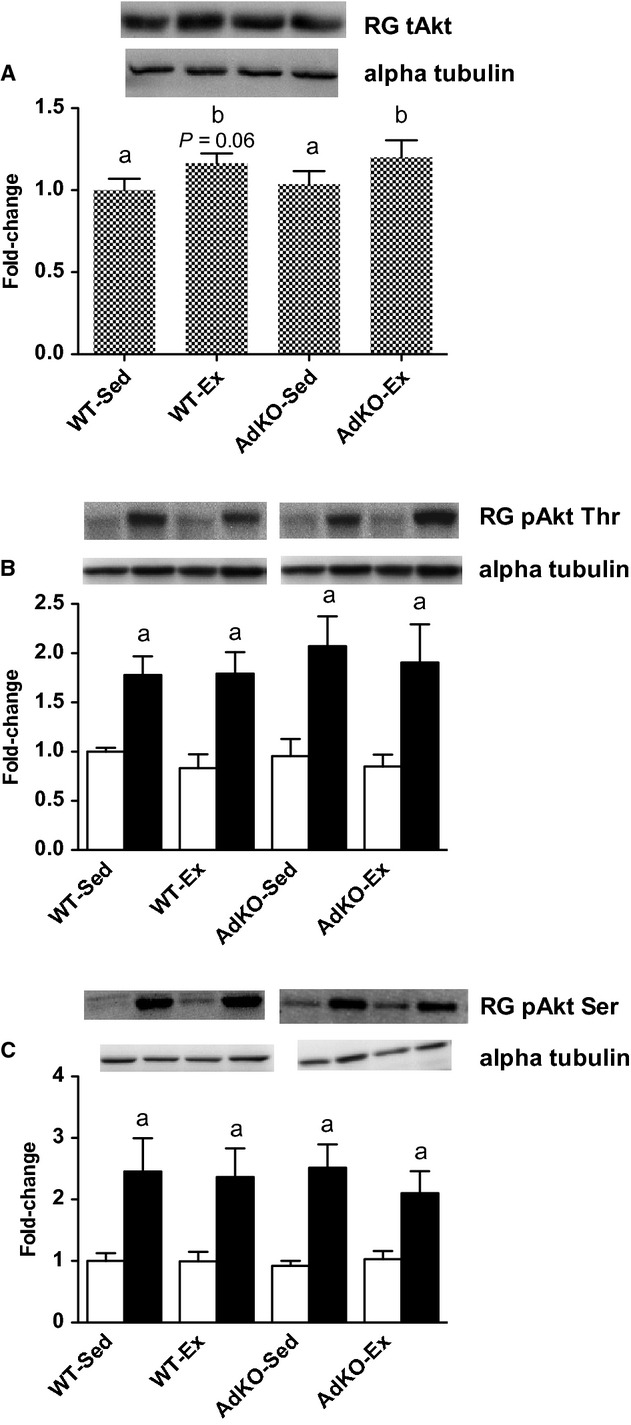
Western blots of total AKT protein content (A) and basal (open bars) and insulin‐stimulated (filled bars) phosphorylation of AKT at threonine 308 (B) and serine 473 (C) from red gastrocnemius muscle. Data are expressed relative to basal WT‐Sed group, mean ± SE,* n* = 8–10. Experimental groups not sharing a letter are significantly different, *P *< 0.05. WT‐Sed, wild‐type+no exercise; WT‐Ex, wild‐type+exercise training; AdKO‐Sed, Ad knockout+no exercise; Ad‐Ex, Ad knockout+exercise training.

There were no differences in total AKT content between genotypes in sedentary or exercise‐trained mice in WG (Fig. 3). There was an overall exercise training response for AKT in RG (*P* < 0.05); however, the training effect did not reach significance in either genotype (WT, +16 ± 5%, *P* = 0.06; AdKO, +16 ± 8%, *P* = 0.06; Fig. 4). Insulin significantly increased the phosphorylation of AKT at serine 473 and threonine 308 in RG and WG with no apparent effect of genotype or exercise training (Figs. 3, 4).

**Figure 5.**
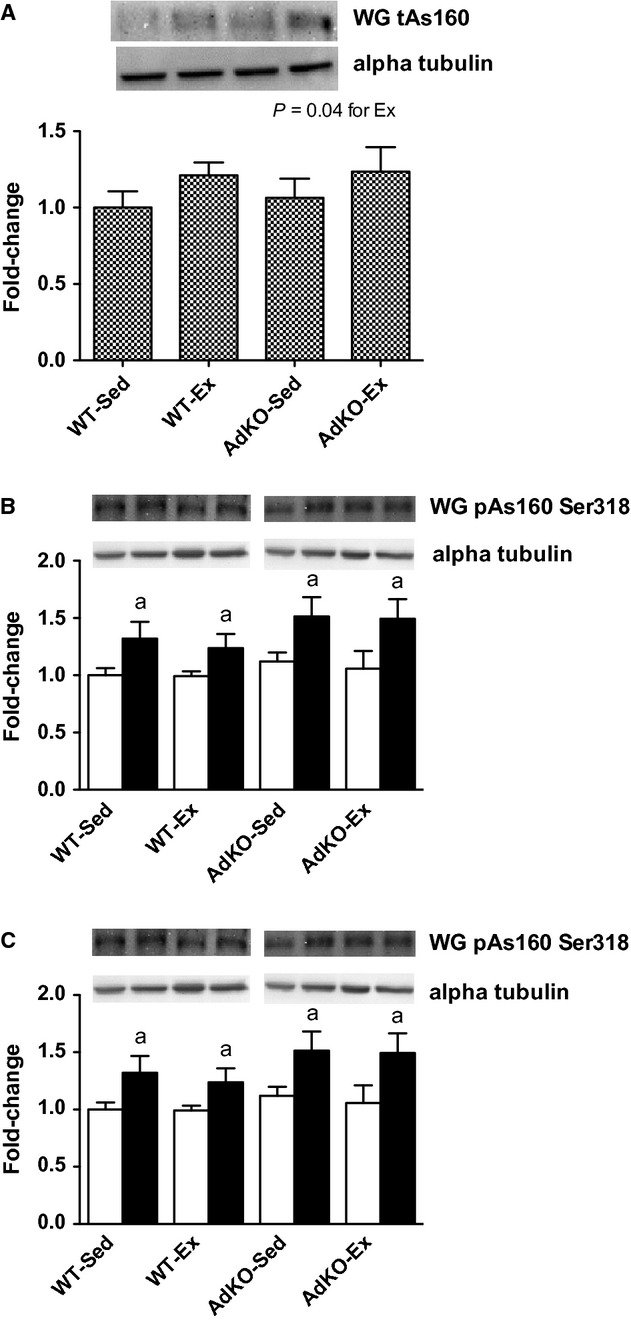
Western blots of total AS160 protein content (A) and basal (open bars) and insulin‐stimulated (filled bars) phosphorylation of AKT at serine 318 (B) and serine 588 (C) from white gastrocnemius muscle. Data are expressed relative to basal WT‐Sed group, mean ± SE,* n* = 8–10. Experimental groups not sharing a letter are significantly different, *P *< 0.05. WT‐Sed, wild‐type+no exercise; WT‐Ex, wild‐type+exercise training; AdKO‐Sed, Ad knockout+no exercise; Ad‐Ex, Ad knockout+exercise training.

**Figure 6.**
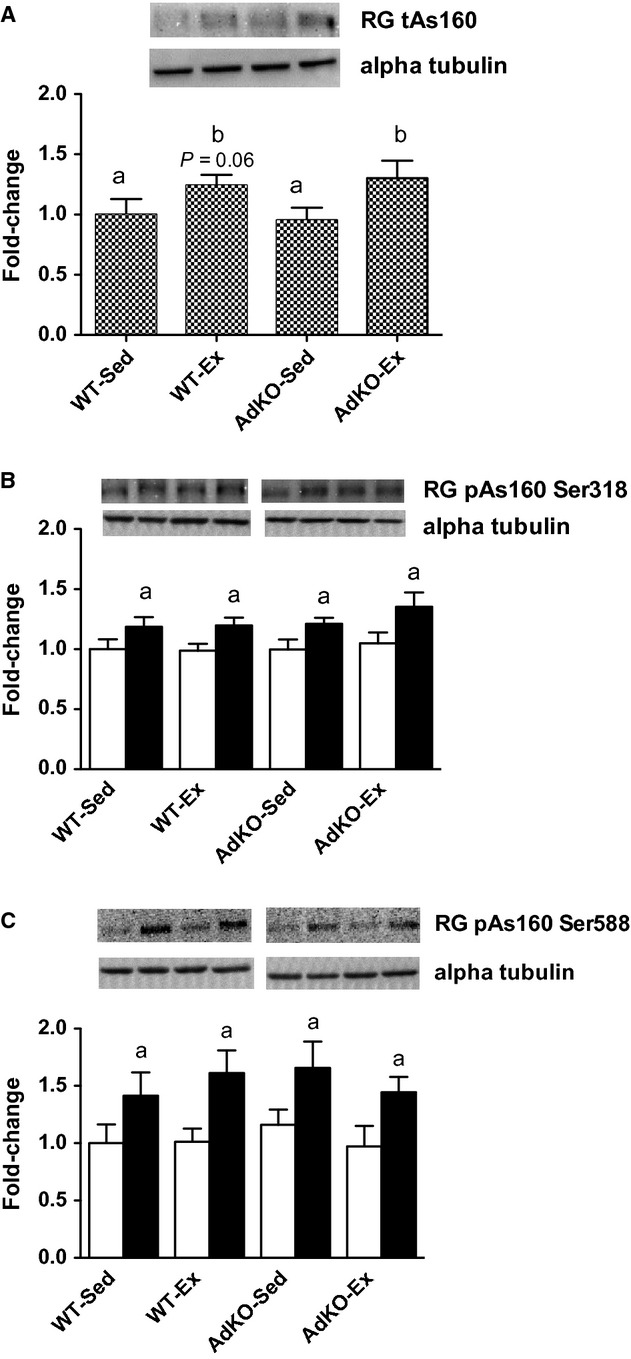
Western blots of total AS160 protein content (A) and basal (open bars) and insulin‐stimulated (filled bars) phosphorylation of AKT at serine 318 (B) and serine 588 (C) from red gastrocnemius muscle. Data are expressed relative to basal WT‐Sed group, mean ± SE,* n* = 8–10. Experimental groups not sharing a letter are significantly different, *P *< 0.05. WT‐Sed, wild‐type+no exercise; WT‐Ex, wild‐type+exercise training; AdKO‐Sed, Ad knockout+no exercise; Ad‐Ex, Ad knockout+exercise training.

Total AS160 content was not significantly different between WT and AdKO mice in sedentary or exercise‐trained groups. There was an overall exercise response in WG (*P* < 0.05; Fig. 5), although there was no significant effect of exercise training within either genotype. In RG, exercise training increased total AS160 in AdKO mice (+44 ± 8%, *P* < 0.05; Fig. 6). This tended to also be true for WT mice, but did not reach significance (+28 ± 7%, *P* = 0.06). Insulin increases the phosphorylation of AS160 at serine 318 and 588 in RG and WG in all experimental groups. There were no effects of genotype or exercise training on the phosphorylation of AS160 in either RG or WG (Figs 5, 6).

### GLUT4 and FAT/CD36 protein content

**Figure 7.**
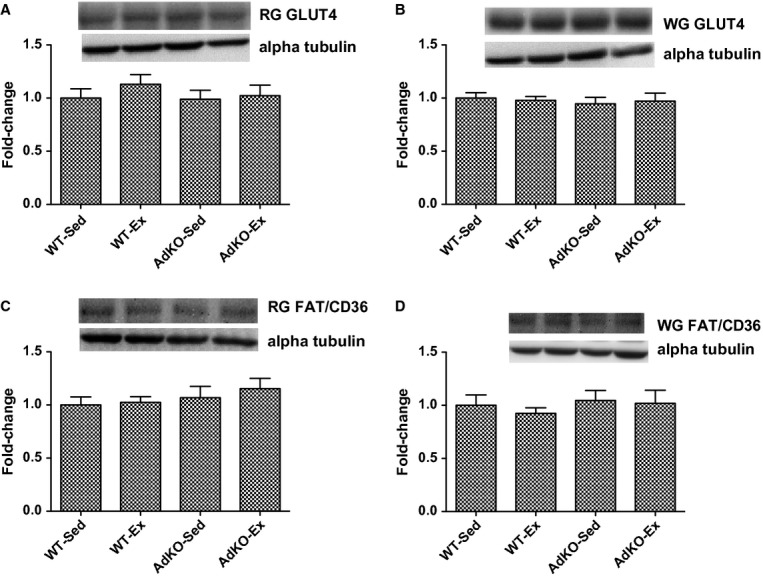
Western blots of total GLUT4 (A, B) and FAT/CD36 (C, D) protein content from whole red (A, C) and white (B, D) gastrocnemius muscle. Data are expressed relative to WT‐Sed group, mean ± SE,* n* = 8–10. Experimental groups not sharing a letter are significantly different, *P* < 0.05. WT‐Sed, wild‐type+no exercise; WT‐Ex, wild‐type+exercise training; AdKO‐Sed, Ad knockout+no exercise; Ad‐Ex, Ad knockout+exercise training.

Whole muscle GLUT4 and FAT/CD36 protein from red or white gastrocnemius was not different between groups (Fig. 7).

## Discussion

It is generally believed that Ad functions as an insulin sensitizer. Acute Ad treatment directly increases glucose transport in muscle cells (Yamauchi et al. 2002; Mao et al. 2006; Wang et al. 2007) and reduces hepatic glucose production (Berg et al. 2001; Combs et al. 2001). Chronic Ad treatment or overexpression of Ad results in a robust improvement in insulin sensitivity in lean, obese, and diet‐induced obese mice (Yamauchi et al. 2001; Kandasamy et al. 2012; Vu et al. 2013). This is mediated by a combination of reduced hepatic gluconeogenic enzymes (PEPCK and G6Pase) (Berg et al. 2001; Combs et al. 2001; Yamauchi et al. 2002) and increased oxidative gene expression skeletal muscle (Yamauchi et al. 2001). Recent findings demonstrate that in muscle cells, Ad activates Ca2+/AMPK pathways and PGC1alpha (Iwabu et al. 2010), which are critical signaling events in response to contraction that are implicated with exercise adaptations, including improvements in insulin sensitivity (McKinsey et al. 2000; Michael et al. 2001; Ojuka et al. 2002; Holmes et al. 2005; McGee et al. 2008; Richter and Hargreaves 2013). Given that circulating Ad has been reported to be increased by chronic exercise (Kriketos et al. 2004), it is reasonable to hypothesize that Ad may be in part responsible for training‐induced improvements in glucose and insulin tolerance. Indeed, TZDs have been shown to be relatively ineffective at improving glucose tolerance in the absence of Ad (Kubota et al. 2006). Here, we examined whether Ad plays a role in the ability of endurance exercise training to improve glucose/insulin tolerance and insulin signaling using a commercially available AdKO mouse model. We find that 8 weeks of treadmill running improved glucose and insulin tolerance in the absence of Ad, and to a similar extent that was observed in WT mice. Furthermore, while the exercise‐effect on insulin signaling protein content was modest, there were no apparent differences between WT and AdKO mice. Therefore, our results do not support the hypothesis that Ad is required for exercise‐mediated improvements in glucose homeostasis.

Recent attempts at delineating the Ad signaling mechanisms have revealed substantial overlap with those pathways activated during skeletal muscle contraction. These include the activation of CAMKKB and AMPK, and the upregulation of PGC1a, events by which exercise purportedly increases skeletal muscle oxidative capacity and insulin response (Iwabu et al. 2010). In addition, circulating Ad has been reported to increase following regular exercise (Kriketos et al. 2004). Collectively, this suggests that Ad could, at least in part, mediate the insulin‐sensitizing effects of exercise training. Here, we find no detriment to exercise‐mediated improvements in glucose and insulin tolerance in the absence of Ad. In fact, we report a small but significant elevation in insulin tolerance in AdKO compared to WT mice with endurance exercise training.

### Changes in insulin signaling

There is inconsistency in the literature in terms of the response of skeletal muscle insulin signaling to endurance exercise training. Some studies report increases in insulin receptor tyrosine phosphorylation, IRS1/2‐associated PI3K activity, and AKT phosphorylation in response to endurance exercise (Chibalin et al. 2000; Kump and Booth 2005). Many, however, report that exercise training improves insulin‐stimulated glucose transport in rodents and humans in conjunction with increased total content of insulin signaling proteins in rodents and humans (Wojtaszewski et al. 2000; Jessen et al. 2003; Frosig et al. 2007; Farias et al. 2012), but without increasing phosphorylation of IRS1, AKT, AS160, or IRS1‐associated PI3K activity. Our findings are in agreement with the latter, as we do not show greater phosphorylation of AKT or AS160 in response to training. However, it should also be pointed out that we assessed phosphorylation of these proteins 15 min after injection of an insulin dose that should elicit a maximal response. Therefore, it is possible that a maximal response was unaltered by training, and that a lower dose of insulin might have revealed a training effect on insulin sensitivity. Nevertheless, there was absolutely no impairment in insulin‐stimulated phosphorylation of AKT or AS160 in the absence of Ad in muscle from either sedentary or exercise‐trained mice when compared to WT animals. Finally, we do report an exercise response with respect to total AKT and AS160 content in RG in both WT and AdKO mice. In agreement with our insulin tolerance data, this response is actually greater in the muscle of AdKO mice. Collectively, then, our data show no evidence of impaired insulin signaling in trained or untrained muscle in the absence of circulating Ad.

### Conflicting findings from AdKO animals

Our data are surprising given the large body of in vitro and in vivo evidence that indicate a critical insulin‐sensitizing role for Ad. Several studies have reported normal or only modestly impaired metabolism in AdKO mice. Yano et al. (2008) and Nawrocki et al. (2006) both report moderate whole‐body insulin resistance in chow‐fed AdKO mice. Others, however, report normal insulin and glucose tolerance in AdKO mice when compared to WT animals (Ma et al. 2002; Ritchie et al. 2014). Our results support and build upon the latter, demonstrating that the absence of Ad does not impair exercise‐induced increases in insulin sensitivity. We have recently shown that WT and AdKO mice are equally able to increase the gene expression and protein content of mitochondrial proteins in response to acute exercise and exercise training (Ritchie et al. 2014). Collectively, this suggests that the presence of Ad is unnecessary for adaptations to exercise training, at least in lean, chow‐fed mice.

These challenging and conflicting results are difficult to explain. Previous work has demonstrated the impact of genetic background on the severity lipidemia, glycemia, and insulin resistance in transgenic mice. (Colombo et al. 2003; Haluzik et al. 2004) Interestingly, both lipoatrophic AZIP and leptin‐deficient ob/ob mice with a C57BL/6J background are less insulin resistant compared to mice with a FVB background (Colombo et al. 2003; Haluzik et al. 2004). Therefore, although genetic background does not appear to explain the controversial results in AdKO studies (most groups have used a C57BL/6J background (Ma et al. 2002; Maeda et al. 2002; Nawrocki et al. 2006; Ritchie et al. 2014)), it may, at least in part, explain why AdKO mice appear to be metabolically normal, or at worse, only moderately insulin resistant. It should be recognized that the commercially provided knockout and wild‐type mice were of somewhat different genetic backgrounds. that is, wild type were pure BL6 and the KOs were a mixed BL6/sv129. Therefore, while we do not think that the backgrounds likely contribute to our findings, we cannot rule out the possibility that differences in background affected the response of the groups to the exercise.

### Unexpected findings – possibility of compensation?

In contrast to AdKO mice, AdipoR1 receptor KO mice demonstrate a phenotype that one would expect in the absence of Ad signaling (i.e., reduced muscle mitochondria, reduced muscle FA oxidation, and insulin resistance) (Yamauchi et al. 2007; Iwabu et al. 2010). The discrepancy suggests the presence of a compensatory factor that is active in the absence of Ad, but not in the absence of the AdipoR1.

C1q/TNF‐related proteins (CTRP) 1–10 are a recently discovered family of adipokines, which are structurally similar to Ad. Circulating CTRP1 and 3 are reduced in HF diet fed and obese (ob/ob) mice (Peterson et al. 2010, 2012). In vivo and in vitro, CTRP3 reduces gluconeogenesis and gluconeogenic enzyme expression (PEPCK, G6Pase) (Peterson et al. 2010) while CTRP1 overexpression improves glucose and insulin tolerance (Peterson et al. 2012). In endothelial cells CTRP9 activates AMPK via AdipoR1 (Zheng et al. 2011) and, like Ad, acute treatment with CTRP1 increases the phosphorylation of AMPK and ACC and increases FA oxidation in isolated skeletal muscle (Peterson et al. 2012). Importantly, CPTR1 and 6 are increased in AdKO mice (Wong et al. 2008). Therefore, it is tempting to speculate that CPTR proteins may compensate for the absence of Ad in AdKO mice and explain the modest or moderate phenotype observed here and elsewhere (Ma et al. 2002; Nawrocki et al. 2006; Yano et al. 2008). However, our understanding of the function and mechanisms of these signaling molecules is still in its infancy.

### Perspectives and significance

Collectively, our results demonstrate that the absence of Ad does not impair the capacity of endurance exercise training to increase glucose and insulin tolerance in AdKO mice. Instead, insulin tolerance is actually enhanced in AdKO mice following exercise, compared to wild‐type mice. In addition, there were no impairments in insulin signaling or in the protein content of AKT or AS160. Taken together with previous findings, our data indicate that AdKO mice may have sufficient compensations to override the absence of Ad. Nevertheless, we have demonstrated that Ad per se is not required for exercise‐induced adaptations in mitochondrial content or improvements in glucose and insulin tolerance.

## Acknowledgments

The authors wish to thank Leisha Klinger and Melissa Hamilton for their technical assistance with the oral tolerance tests and exercise training.

## Conflict of Interest

None declared.

## References

[b1] Arita Y. Kihara S. Ouchi N. Takahashi M. Maeda K. Miyagawa J. 1999 Paradoxical decrease of an adipose‐specific protein, adiponectin, in obesity. Biochem. Biophys. Res. Commun.; 257:79-83. 10092513 10.1006/bbrc.1999.0255

[b2] Berg A. H. Combs T. P. Du X. Brownlee M. Scherer P. E. 2001 The adipocyte‐secreted protein Acrp30 enhances hepatic insulin action. Nat. Med.; 7:947-953. 11479628 10.1038/90992

[b3] Bruce C. R. Anderson M. J. Carey A. L. Newman D. G. Bonen A. Kriketos A. D. 2003 Muscle oxidative capacity is a better predictor of insulin sensitivity than lipid status. J. Clin. Endocrinol. Metab.; 88:5444-5451. 14602787 10.1210/jc.2003-030791

[b4] Bruce C. R. Mertz V. A. Heigenhauser G. J. Dyck D. J. 2005 The stimulatory effect of globular adiponectin on insulin‐stimulated glucose uptake and fatty acid oxidation is impaired in skeletal muscle from obese subjects. Diabetes; 54:3154-3160. 16249439 10.2337/diabetes.54.11.3154

[b5] Cartee G. D. Holloszy J. O. 1990 Exercise increases susceptibility of muscle glucose transport to activation by various stimuli. Am. J. Physiol.; 258:E390-E393. 2305881 10.1152/ajpendo.1990.258.2.E390

[b6] Cartee G. D. Young D. A. Sleeper M. D. Zierath J. Wallberg‐Henriksson H. Holloszy J. O. 1989 Prolonged increase in insulin‐stimulated glucose transport in muscle after exercise. Am. J. Physiol.; 256:E494-E499. 2650561 10.1152/ajpendo.1989.256.4.E494

[b7] Chibalin A. V. Yu M. Ryder J. W. Song X. M. Galuska D. Krook A. 2000 Exercise‐induced changes in expression and activity of proteins involved in insulin signal transduction in skeletal muscle: differential effects on insulin‐receptor substrates 1 and 2. Proc. Natl Acad. Sci. USA; 97:38-43. 10618367 10.1073/pnas.97.1.38 PMC26612

[b8] Colombo C. Haluzik M. Cutson J. J. Dietz K. R. Marcus‐Samuels B. Vinson C. 2003 Opposite effects of background genotype on muscle and liver insulin sensitivity of lipoatrophic mice. Role of triglyceride clearance. J. Biol. Chem.; 278:3992-3999. 12456680 10.1074/jbc.M207665200

[b9] Combs T. P. Berg A. H. Obici S. Scherer P. E. Rossetti L. 2001 Endogenous glucose production is inhibited by the adipose‐derived protein Acrp30. J. Clin. Invest.; 108:1875-1881. 11748271 10.1172/JCI14120 PMC209474

[b10] Farias J. M. Maggi R. M. Tromm C. B. Silva L. A. Luciano T. F. Marques S. O. 2012 Exercise training performed simultaneously to a high‐fat diet reduces the degree of insulin resistance and improves adipoR1‐2/APPL1 protein levels in mice. Lipids Health Dis.; 11:134 23046739 10.1186/1476-511X-11-134 PMC3539884

[b11] Frosig C. Rose A. J. Treebak J. T. Kiens B. Richter E. A. Wojtaszewski J. F. 2007 Effects of endurance exercise training on insulin signaling in human skeletal muscle: interactions at the level of phosphatidylinositol 3‐kinase, Akt, and AS160. Diabetes; 56:2093-2102. 17513702 10.2337/db06-1698

[b12] Fruebis J. Tsao T. S. Javorschi S. Ebbets‐Reed D. Erickson M. R. Yen F. T. 2001 Proteolytic cleavage product of 30‐kDa adipocyte complement‐related protein increases fatty acid oxidation in muscle and causes weight loss in mice. Proc. Natl Acad. Sci. USA; 98:2005-2010. 11172066 10.1073/pnas.041591798 PMC29372

[b13] Gao J. Gulve E. A. Holloszy J. O. 1994 Contraction‐induced increase in muscle insulin sensitivity: requirement for a serum factor. Am. J. Physiol.; 266:E186-E192. 8141276 10.1152/ajpendo.1994.266.2.E186

[b14] Haluzik M. Colombo C. Gavrilova O. Chua S. Wolf N. Chen M. 2004 Genetic background (C57BL/6J versus FVB/N) strongly influences the severity of diabetes and insulin resistance in ob/ob mice. Endocrinology; 145:3258-3264. 15059949 10.1210/en.2004-0219

[b15] Holmes B. F. Sparling D. P. Olson A. L. Winder W. W. Dohm G. L. 2005 Regulation of muscle GLUT4 enhancer factor and myocyte enhancer factor 2 by AMP‐activated protein kinase. Am. J. Physiol. Endocrinol. Metab.; 289:E1071-E1076. 16105857 10.1152/ajpendo.00606.2004

[b16] Host H. H. Hansen P. A. Nolte L. A. Chen M. M. Holloszy J. O. 1998 Rapid reversal of adaptive increases in muscle GLUT‐4 and glucose transport capacity after training cessation. J. Appl. Physiol.; 84:798-802. 9480935 10.1152/jappl.1998.84.3.798

[b17] Hotta K. Funahashi T. Bodkin N. L. Ortmeyer H. K. Arita Y. Hansen B. C. 2001 Circulating concentrations of the adipocyte protein adiponectin are decreased in parallel with reduced insulin sensitivity during the progression to type 2 diabetes in rhesus monkeys. Diabetes; 50:1126-1133. 11334417 10.2337/diabetes.50.5.1126

[b18] Iwabu M. Yamauchi T. Okada‐Iwabu M. Sato K. Nakagawa T. Funata M. 2010 Adiponectin and AdipoR1 regulate PGC‐1alpha and mitochondria by Ca(2+) and AMPK/SIRT1. Nature; 464:1313-1319. 20357764 10.1038/nature08991

[b19] Jessen N. Pold R. Buhl E. S. Jensen L. S. Schmitz O. Lund S. 2003 Effects of AICAR and exercise on insulin‐stimulated glucose uptake, signaling, and GLUT‐4 content in rat muscles. J. Appl. Physiol.; 94:1373-1379. 12496137 10.1152/japplphysiol.00250.2002

[b20] Kandasamy A. D. Sung M. M. Boisvenue J. J. Barr A. J. Dyck J. R. 2012 Adiponectin gene therapy ameliorates high‐fat, high‐sucrose diet‐induced metabolic perturbations in mice. Nutr. Diabetes; 2:e45 23446660 10.1038/nutd.2012.18 PMC3461354

[b21] Kriketos A. D. Gan S. K. Poynten A. M. Furler S. M. Chisholm D. J. Campbell L. V. 2004 Exercise increases adiponectin levels and insulin sensitivity in humans. Diabetes Care; 27:629-630. 14747265 10.2337/diacare.27.2.629

[b22] Kubota N. Terauchi Y. Kubota T. Kumagai H. Itoh S. Satoh H. 2006 Pioglitazone ameliorates insulin resistance and diabetes by both adiponectin‐dependent and ‐independent pathways. J. Biol. Chem.; 281:8748-8755. 16431926 10.1074/jbc.M505649200

[b23] Kump D. S. Booth F. W. 2005 Alterations in insulin receptor signalling in the rat epitrochlearis muscle upon cessation of voluntary exercise. J. Physiol.; 562:829-838. 15550465 10.1113/jphysiol.2004.073593 PMC1665545

[b24] Li L. Pan R. Li R. Niemann B. Aurich A. C. Chen Y. 2011 Mitochondrial biogenesis and peroxisome proliferator‐activated receptor‐gamma coactivator‐1alpha (PGC‐1alpha) deacetylation by physical activity: intact adipocytokine signaling is required. Diabetes; 60:157-167. 20929977 10.2337/db10-0331 PMC3012167

[b25] Liu L. Zhang Y. Chen N. Shi X. Tsang B. Yu Y. H. 2007 Upregulation of myocellular DGAT1 augments triglyceride synthesis in skeletal muscle and protects against fat‐induced insulin resistance. J. Clin. Invest.; 117:1679-1689. 17510710 10.1172/JCI30565 PMC1866250

[b26] Ma K. Cabrero A. Saha P. K. Kojima H. Li L. Chang B. H. 2002 Increased beta ‐oxidation but no insulin resistance or glucose intolerance in mice lacking adiponectin. J. Biol. Chem.; 277:34658-34661. 12151381 10.1074/jbc.C200362200

[b27] Maeda N. Shimomura I. Kishida K. Nishizawa H. Matsuda M. Nagaretani H. 2002 Diet‐induced insulin resistance in mice lacking adiponectin/ACRP30. Nat. Med.; 8:731-737. 12068289 10.1038/nm724

[b28] Mao X. Kikani C. K. Riojas R. A. Langlais P. Wang L. Ramos F. J. 2006 APPL1 binds to adiponectin receptors and mediates adiponectin signalling and function. Nat. Cell Biol.; 8:516-523. 16622416 10.1038/ncb1404

[b29] McGee S. L. van Denderen B. J. Howlett K. F. Mollica J. Schertzer J. D. Kemp B. E. 2008 AMP‐activated protein kinase regulates GLUT4 transcription by phosphorylating histone deacetylase 5. Diabetes; 57:860-867. 18184930 10.2337/db07-0843

[b30] McKinsey T. A. Zhang C. L. Olson E. N. 2000 Activation of the myocyte enhancer factor‐2 transcription factor by calcium/calmodulin‐dependent protein kinase‐stimulated binding of 14‐3‐3 to histone deacetylase 5. Proc. Natl Acad. Sci. USA; 97:14400-14405. 11114197 10.1073/pnas.260501497 PMC18930

[b31] Michael L. F. Wu Z. Cheatham R. B. Puigserver P. Adelmant G. Lehman J. J. 2001 Restoration of insulin‐sensitive glucose transporter (GLUT4) gene expression in muscle cells by the transcriptional coactivator PGC‐1. Proc. Natl Acad. Sci. USA; 98:3820-3825. 11274399 10.1073/pnas.061035098 PMC31136

[b32] Nawrocki A. R. Rajala M. W. Tomas E. Pajvani U. B. Saha A. K. Trumbauer M. E. 2006 Mice lacking adiponectin show decreased hepatic insulin sensitivity and reduced responsiveness to peroxisome proliferator‐activated receptor gamma agonists. J. Biol. Chem.; 281:2654-2660. 16326714 10.1074/jbc.M505311200

[b33] Ojuka E. O. Jones T. E. Nolte L. A. Chen M. Wamhoff B. R. Sturek M. 2002 Regulation of GLUT4 biogenesis in muscle: evidence for involvement of AMPK and Ca(2+). Am. J. Physiol. Endocrinol. Metab.; 282:E1008-E1013. 11934664 10.1152/ajpendo.00512.2001

[b34] Peterson J. M. Wei Z. Wong G. W. 2010 C1q/TNF‐related protein‐3 (CTRP3), a novel adipokine that regulates hepatic glucose output. J. Biol. Chem.; 285:39691-39701. 20952387 10.1074/jbc.M110.180695 PMC3000950

[b35] Peterson J. M. Aja S. Wei Z. Wong G. W. 2012 CTRP1 protein enhances fatty acid oxidation via AMP‐activated protein kinase (AMPK) activation and acetyl‐CoA carboxylase (ACC) inhibition. J. Biol. Chem.; 287:1576-1587. 22086915 10.1074/jbc.M111.278333 PMC3256898

[b36] Richter E. A. Hargreaves M. 2013 Exercise, GLUT4, and skeletal muscle glucose uptake. Physiol. Rev.; 93:993-1017. 23899560 10.1152/physrev.00038.2012

[b37] Ritchie I. R. Macdonald T. L. Wright D. C. Dyck D. J. 2014 Adiponectin is sufficient, but not required, for exercise‐induced increases in the expression of skeletal muscle mitochondrial enzymes. J. Physiol.; 592:2653-2665. 24687585 10.1113/jphysiol.2014.273680 PMC4080944

[b38] Rose A. J. Hargreaves M. 2003 Exercise increases Ca2+‐calmodulin‐dependent protein kinase II activity in human skeletal muscle. J. Physiol.; 553:303-309. 14565989 10.1113/jphysiol.2003.054171 PMC2343484

[b39] Schenk S. Horowitz J. F. 2007 Acute exercise increases triglyceride synthesis in skeletal muscle and prevents fatty acid‐induced insulin resistance. J. Clin. Invest.; 117:1690-1698. 17510709 10.1172/JCI30566 PMC1866251

[b40] Smith J. A. Collins M. Grobler L. A. Magee C. J. Ojuka E. O. 2007 Exercise and CaMK activation both increase the binding of MEF2A to the Glut4 promoter in skeletal muscle in vivo. Am. J. Physiol. Endocrinol. Metab.; 292:E413-E420. 16985263 10.1152/ajpendo.00142.2006

[b41] Sriwijitkamol A. Coletta D. K. Wajcberg E. Balbontin G. B. Reyna S. M. Barrientes J. 2007 Effect of acute exercise on AMPK signaling in skeletal muscle of subjects with type 2 diabetes: a time‐course and dose‐response study. Diabetes; 56:836-848. 17327455 10.2337/db06-1119 PMC2844111

[b42] Vu V. Liu Y. Sen S. Xu A. Sweeney G. 2013 Delivery of adiponectin gene to skeletal muscle using ultrasound targeted microbubbles improves insulin sensitivity and whole body glucose homeostasis. Am. J. Physiol. Endocrinol. Metab.; 304:E168-E175. 23132298 10.1152/ajpendo.00493.2012 PMC3543570

[b43] Wallberg‐Henriksson H. Constable S. H. Young D. A. Holloszy J. O. 1988 Glucose transport into rat skeletal muscle: interaction between exercise and insulin. J. Appl. Physiol.; 65:909-913. 3049515 10.1152/jappl.1988.65.2.909

[b44] Wang C. Mao X. Wang L. Liu M. Wetzel M. D. Guan K. L. 2007 Adiponectin sensitizes insulin signaling by reducing p70 S6 kinase‐mediated serine phosphorylation of IRS‐1. J. Biol. Chem.; 282:7991-7996. 17244624 10.1074/jbc.M700098200

[b45] Winder W. W. Holmes B. F. Rubink D. S. Jensen E. B. Chen M. Holloszy J. O. 2000 Activation of AMP‐activated protein kinase increases mitochondrial enzymes in skeletal muscle. J. Appl. Physiol.; 88:2219-2226. 10846039 10.1152/jappl.2000.88.6.2219

[b46] Wojtaszewski J. F. Hansen B. F. Gade Kiens B. Markuns J. F. Goodyear L. J. 2000 Insulin signaling and insulin sensitivity after exercise in human skeletal muscle. Diabetes; 49:325-331. 10868952 10.2337/diabetes.49.3.325

[b47] Wong G. W. Krawczyk S. A. Kitidis‐Mitrokostas C. Revett T. Gimeno R. Lodish H. F. 2008 Molecular, biochemical and functional characterizations of C1q/TNF family members: adipose‐tissue‐selective expression patterns, regulation by PPAR‐gamma agonist, cysteine‐mediated oligomerizations, combinatorial associations and metabolic functions. Biochem. J.; 416:161-177. 18783346 10.1042/BJ20081240 PMC3936483

[b48] Wright D. C. Geiger P. C. Han D. H. Jones T. E. Holloszy J. O. 2007 Calcium induces increases in peroxisome proliferator‐activated receptor gamma coactivator‐1alpha and mitochondrial biogenesis by a pathway leading to p38 mitogen‐activated protein kinase activation. J. Biol. Chem.; 282:18793-18799. 17488713 10.1074/jbc.M611252200

[b49] Yamauchi T. Kamon J. Waki H. Terauchi Y. Kubota N. Hara K. 2001 The fat‐derived hormone adiponectin reverses insulin resistance associated with both lipoatrophy and obesity. Nat. Med.; 7:941-946. 11479627 10.1038/90984

[b50] Yamauchi T. Kamon J. Minokoshi Y. Ito Y. Waki H. Uchida S. 2002 Adiponectin stimulates glucose utilization and fatty‐acid oxidation by activating AMP‐activated protein kinase. Nat. Med.; 8:1288-1295. 12368907 10.1038/nm788

[b51] Yamauchi T. Nio Y. Maki T. Kobayashi M. Takazawa T. Iwabu M. 2007 Targeted disruption of AdipoR1 and AdipoR2 causes abrogation of adiponectin binding and metabolic actions. Nat. Med.; 13:332-339. 17268472 10.1038/nm1557

[b52] Yano W. Kubota N. Itoh S. Kubota T. Awazawa M. Moroi M. 2008 Molecular mechanism of moderate insulin resistance in adiponectin‐knockout mice. Endocr. J.; 55:515-522. 18446001 10.1507/endocrj.k08e-093

[b53] Zheng Q. Yuan Y. Yi W. Lau W. B. Wang Y. Wang X. 2011 C1q/TNF‐related proteins, a family of novel adipokines, induce vascular relaxation through the adiponectin receptor‐1/AMPK/eNOS/nitric oxide signaling pathway. Arterioscler. Thromb. Vasc. Biol.; 31:2616-2623. 21836066 10.1161/ATVBAHA.111.231050 PMC3197867

